# Chronic Liver Diseases: What is Up?

**DOI:** 10.3390/jcm13020613

**Published:** 2024-01-22

**Authors:** Sharmila Fagoonee, Pietro Invernizzi

**Affiliations:** 1 Institute of Biostructure and Bioimaging (CNR), Molecular Biotechnology Center “Guido Tarone”, Via Nizza 52, 10126 Turin, Italy; 2 Division of Gastroenterology, Center for Autoimmune Liver Diseases, Department of Medicine and Surgery, University of Milano-Bicocca, 20126 Monza, Italy; 3 European Reference Network on Hepatological Diseases (ERN RARE-LIVER), IRCSS Fondazione San Gerardo dei Tintori, 20900 Monza, Italy

During the preparation of this Special Issue, Dr. Rinaldo Pellicano, our co-guest editor and Gastroenterologist at the Hospitals “Città della Salute e delle Scienze” (Molinette) and “San Giovanni Antica Sede” in Turin, Italy, passed away prematurely. He devoted his research to diagnosing and treating various gastrointestinal and hepatic diseases, with a primary focus on the management of Helicobacter pylori infection. He co-authored numerous scientific papers and books. Dr. Pellicano will be remembered for his exceptional medical expertise and communication skills.

The articles published in this Special Issue cover various stages of chronic liver diseases, ranging from fibrosis to carcinoma. The term “fibrosis” was coined in 1871 by combining the Latin word “fibra,” meaning “fiber,” and the Greek suffix “-osis,” denoting the “state of a disease” (source: https://www.etymonline.com/word/fibrosis, accessed on 8 January 2024). Today, fibrosis is commonly used to describe scarring that occurs as a pathological response to injury or damage during wound healing or repair. If the damage is unchecked, significant tissue remodeling may occur, leading to permanent scarring that can compromise the functionality of the affected organ.

The liver, the largest internal organ of the body, plays a crucial role in numerous essential functions, including the control of homeostasis, metabolism, immunity, digestion, detoxification, and vitamin storage [1]. Liver fibrosis results from a variety of chronic insults, such as exposure to toxins or drugs, alcohol abuse, infections of parasitic or viral nature, cholestasis, as well as metabolic and genetic diseases [2]. With the success of therapies and vaccination strategies against hepatitis viruses, excessive nutrition has emerged as the primary contemporary threat to a healthy liver, making non-alcoholic fatty liver disease the most encountered hepatic disorder of the century [3]. When the liver’s extraordinary capacity for tissue repair and regeneration is overwhelmed, liver fibrosis, the common precursor to cirrhosis and carcinoma, develops. Detecting liver fibrosis early significantly reduces morbidity and mortality, representing a significant challenge for researchers and hepatologists [4]. Growing evidence suggests that even in the advanced stages of the disease, effective treatment for the underlying insult can lead to the histological and biomarker-wise disappearance of fibrotic lesions.

We are increasingly aware that liver fibrosis exhibits distinct patterns of evolution and is influenced by various underlying mechanisms, depending on its etiology. This emphasizes the need to thoroughly dissect these physiopathological pathways, including transcriptional, post-transcriptional, and epigenetic control of liver fibrosis stage-wise advancement to cirrhosis and carcinoma, to develop specific diagnostic and therapeutic approaches. Such advancements are crucial for enhancing the clinical management of chronic liver diseases, allowing for more targeted and effective interventions [5]. Recently, Artificial Intelligence (AI) has been explored in the development of machine learning and deep learning approaches for early disease diagnosis and management through diagnostic image analysis, biomarker discovery, and predicting personalized clinical outcomes [6]. Further optimization of specificity and accuracy is required before the implementation of AI into daily clinical routine. In the meantime, novel biomarkers or imaging modalities capable of detecting early pathological changes in the liver in a non-invasive way are under scrutiny, and the coming years will witness their integration into clinical settings. 
